# Integrated bioinformatics and machine learning to explore the common mechanisms and potential biomarkers between periodontitis and preterm birth

**DOI:** 10.3389/fcell.2026.1763374

**Published:** 2026-02-20

**Authors:** Feng Mei, Yutong Liu, Wenting Xu, Ruoyun Liu, Xinlin Wang, Tingting Li, Yi Chen, Tingting Wu, Wei Zhang

**Affiliations:** 1 College and Hospital of Stomatology, Anhui Medical University, Anhui Provincial Key Laboratory of Oral Diseases Research, Hefei, China; 2 Department of Obstetrics and Gynaecology, The First Affiliated Hospital of Anhui Medical University, Hefei, Anhui, China

**Keywords:** bioinformatics, biomarkers, immune infiltration, machine learning, periodontitis, preterm birth

## Abstract

**Background:**

There is accumulating evidence suggesting an association between periodontitis (PD) and preterm birth (PTB), but the underlying mechanisms have not been fully elucidated. This study aims to explore potential biomarkers and mechanisms between PD and PTB through integrated bioinformatics and machine learning approaches.

**Methods:**

Datasets for PD (GSE16134 and GSE10334) and PTB (GSE203507, GSE174415, GSE18809, GSE73685 and GSE120480) were acquired from Gene Expression Omnibus (GEO). Then we performed Weighted gene co-expression network analysis (WGCNA), differential expressed genes (DEGs) analysis and three machine learning algorithms to identify cross-talk genes. To evaluate the potential of cross-talk genes as diagnostic biomarkers for PD and PTB, receiver operating characteristic (ROC) curve analysis and expression analysis were conducted. We then conducted functional enrichment analysis to elucidate the biological roles of the common DEGs. Single-sample gene set enrichment analysis (ssGSEA) assessed immune cell patterns of PD and PTB and biomarker-immune cell correlations. Additionally, we constructed a protein-protein interaction (PPI) network and further analyzed potential biomarkers using the cytoHubba plugin in Cytoscape software. Ultimately, the expression of the core genes in the PD animal model were validated.

**Results:**

We identified four cross-talk genes through the integrated analysis. Common DEGs were mainly concentrated in immune-related pathways. Following expression analysis and ROC curve analysis, we identified two genes (CD53 and BIN2) as potential biomarkers for PD and PTB. These genes were upregulated in disease groups compared to controls and exhibited strong diagnostic performance (AUC > 0.7) in both the training and validation cohorts. Moreover, CD53 and BIN2 displayed high connectivity within the PPI network. Immune cell infiltration analysis revealed that multiple immune cell types exhibited consistent upregulation in both diseases. In the PD model, consistent upregulation of CD53 and BIN2 was observed in the maxillary bone.

**Conclusion:**

We identified two potential biomarkers (CD53 and BIN2) for the concurrent diagnosis of PD and PTB, and suggested that the potential common mechanism of these two diseases may be correlated with the immune response. This study provides novel insights into the pathogenesis of both diseases, thereby informing future preventive, diagnostic and therapeutic strategies.

## 1 Introduction

Preterm birth (PTB) is clinically defined as birth prior to 37 weeks of pregnancy (Spada et al., 2020). Globally, about 16% of mortality in children under five and 35% of neonatal mortality are attributable to complications from PTB, making it a primary cause of death in these age groups (Chawanpaiboon et al., 2019). Despite the continuous advancement of research and technology, which has markedly increased survival rates among preterm infants, the health of these infants continues to be threatened by both immediate and long-term complications arising from multisystem immaturity (Lv et al., 2024). These include serious short-term conditions such as acute intracerebral hemorrhage, necrotizing enterocolitis, and respiratory distress syndrome (Romero et al., 2014), and longer-term conditions such as cardiovascular and metabolic diseases (Johansson et al., 2005; Sipola-Leppänen et al., 2015). Such complications are imposing a substantial medical and economic burden on society. Therefore, more and more scholars have been investigating the pathogenesis of PTB to provide new preventive and therapeutic strategies in clinical practice.

Periodontitis (PD) is a chronic inflammatory disease that causes destruction of periodontal supporting structures and tooth loss and potentially contributes to systemic inflammation (Hajishengallis, 2015; Kinane et al., 2017; Mei et al., 2020). It has been estimated that approximately 62% of the adults suffer from PD (Trindade et al., 2023). Therefore, PD is among the most prevalent chronic diseases worldwide (Kassebaum et al., 2014). Epidemiological studies have shown that PD is associated with at least 43 systemic diseases, such as neurodegenerative disorders, diabetes, cardiovascular diseases, and oncology (GBD 2017 Disease and Injury Incidence and Prevalence Collaborators, 2018; Cullinan and Seymour, 2013). Of particular concern is its impact on pregnancy. PD is highly prevalent during pregnancy driven by hormonal fluctuations, affecting 60%–75% of pregnant women. This condition may further contribute to adverse pregnancy outcomes (APOs) (Chen et al., 2022; Daalderop et al., 2018; Raju and Berens, 2021). Consequently, epidemiological studies have demonstrated that PD and APOs are highly positively correlated (Ide and Papapanou, 2013). APOs are detrimental health events that occur during pregnancy or shortly thereafter, impacting maternal, fetal, or neonatal wellbeing, such as PTB, low birth weight, preeclampsia (PE). Therefore, PD in pregnancy has received more attention in the last few years. In particular, a growing number of studies have established a clear association between PD and PTB. Latorre Uriza et al. indicated that PD patients exhibited elevated serum level of inflammatory cytokines such as IL-2, TNF-α and IL-10, suggesting a link to a higher risk of PTB (Latorre et al., 2018). A systematic review also found that maternal PD is linked to a 1.6-fold increased risk of PTB (Daalderop et al., 2018). Furthermore, a meta-analysis demonstrated that periodontal treatment during pregnancy reduced the risk of PTB (Bi et al., 2021).

Substantial evidence has revealed a bidirectional association between PD and PTB. Given the severe health implications and public health challenges resulting from PTB, combined with the significant global burden of PD, it is important to study the common pathological pathways the two diseases are connected. Based on the potential mechanisms linking PD and APOs, it has also been hypothesized that the association between PD and PTB may involve both direct and indirect pathways: the direct mechanism is systemic hematogenous dissemination of oral pathogens which can potentially cause intrauterine infection. But this route may not be predominant; the indirect mechanism is through spread of pro-inflammatory mediators, resulting in systemic inflammation. This route is thought to be the principal driver of PTB. However, the precise mechanism of the association between PD and PTB is still a hypothesis at present and requires additional research and evidence to further elucidate (Figuero et al., 2020; Goldenberg and Culhane, 2006).

The pathological interactions and molecular mechanisms are still not completely understood because of the intricate interrelationship between PD and PTB. Due to the rapid evolution of biotechnological capabilities, bioinformatics has rapidly emerged as an important approach to understand disease pathogenesis and identify new biomarkers. Therefore, we employed an integrated approach combining bioinformatics analysis and machine learning to investigate the overlapping genes and related signaling pathways between PD and PTB. These common genes were further examined using expression level analysis, receiver operating characteristic (ROC) curves, immune cell infiltration analysis, and protein-protein interaction (PPI) networks. Ultimately, we identified two potential biomarkers (CD53 and BIN2) for PD and PTB. And our research is the first to propose their potential utility in diagnosing this condition, thereby providing a new perspective for understanding the interconnected pathogenic mechanisms of PD and PTB. Overall, this study aims to provide insights into their shared pathogenesis by identifying potential biomarkers associated with both PD and PTB and to further seek potential breakthroughs for clinical prevention and treatment strategies.

## 2 Materials and methods

### 2.1 Data acquisition and downloading

**FIGURE 1:**
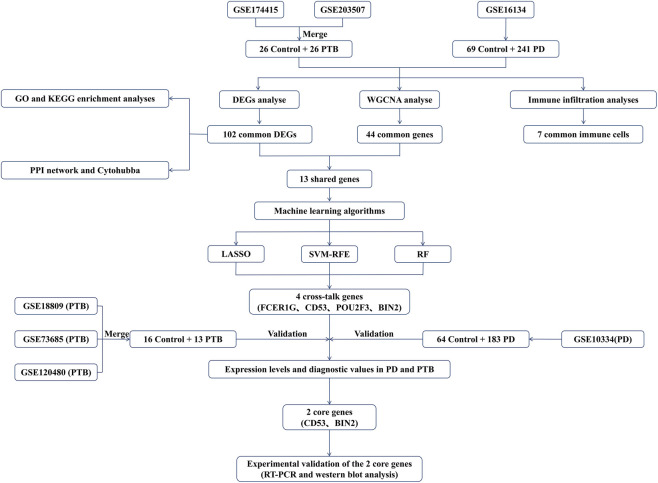
The flow chart for the whole study.

The workflow of this study was presented in the flowchart shown in Figure 1. The PD and PTB datasets analyzed in this study were acquired from the Gene Expression Omnibus (GEO) database (https://www.ncbi.nlm.nih.gov/geo/) (Barrett et al., 2013). The Screening criteria we obtained two PD datasets (GSE16134 and GSE10334) were as follows: 1. All datasets originated from *Homo sapiens*, 2. gene expression profiling was performed using microarray technology, 3. data from control and disease groups were included in the dataset. The screening criteria we obtained five PTB datasets (GSE174415, GSE203507, GSE18809, GSE73685 and GSE120480) were as follows: 1. All datasets originated from *H. sapiens*, 2. the sample cohort included placental or placenta-associated tissue specimens, 3. gene expression profiling was performed using microarray technology, 4. data from control and disease groups were included in the dataset. Table 1 showed details of the datasets.

**TABLE 1:** Basic information of GEO datasets used in the study.

Disease	Accession number	Platform	Samples (disease/control)	Attribute
PD	GSE16134	GPL570	241/69	Train set
	GSE10334	GPL570	183/64	Validation set
PTB	GSE174415	GPL11154	16/16	Train set
	GSE203507	GPL16791	10/10	
	GSE18809	GPL570	5/5	Validation set
	GSE73685	GPL6244	2/5	
	GSE120480	GPL16791	6/6	

### 2.2 Data integration and batch effect correction

The two datasets GSE203507, GSE174415 were extracted and merged. Then, data normalization was performed utilizing the “sva” package (version 3.54.0) in R (version 4.4.3) to correct for batch effects. The integration efficacy was evaluated via principal component analysis (PCA).

### 2.3 Construction of the weighted gene co-expression network analysis (WGCNA) network

We applied the “WGCNA” R package (version 1.73) to identify the gene co-expression modules (Langfelder and Horvath, 2008). All data was processed using RStudio (version 4.4.2), and the WGCNA network was constructed using the approach implemented in the WGCNA R package. Specifically, quality control of the gene expression matrix was carried out with the WGCNA package, and outlier samples were identified through hierarchical clustering to ensure the reliability of data. The pickSoftThreshold function was applied to calculate the scale-free topology fit index and determine an appropriate soft-thresholding power (β-value) meeting scale-free network criteria. Afterward, gene co-expression modules were identified via a one-step network construction method, and a module eigengene clustering dendrogram was generated. We assessed the associations between modules and phenotypic traits to identify the strongest modules. The genes exhibiting the strongest positive and negative correlations were ultimately selected for further analysis. To identify the common genes, the intersection between PD-associated and PTB-associated modules was visualized and extracted using the “VennDiagram” R package (version 1.7.3).

### 2.4 Identification of differentially expressed genes (DEGs)

Following batch effect adjustment, we performed differential expression analysis on the PD and PTB datasets, comparing control and disease groups with the “limma” R package (version 3.62.2). In PD, DEGs were identified by applying thresholds of |log_2_ (fold change)| > 0.5 and P-value < 0.05. In order to identify a sufficient number of DEGs for downstream analysis in PTB, the following criteria were applied: |log_2_ (fold change)| > 0.3 and an adjusted P-value < 0.05. We employed the “ggplot2” (version 3.5.1) and “pheatmap” (version 1.0.12) R packages to visualize DEGs by generating volcano plots and heatmaps. Common DEGs were acquired through a Venn diagram created with the “VennDiagram” package. Common DEGs and the common genes obtained from WGCNA analysis were used to generate a Venn diagram, which identified a set of shared genes.

### 2.5 Machine learning for identification candidate genes

To identify cross-talk genes for PD with PTB, a refined selection of the shared genes was conducted employing three machine-learning algorithms. We employed the LASSO regression algorithm which could improve prediction accuracy via variable selection and regularization. This analysis was implemented in the R software environment, utilizing the “glmnet” package (version 4.1.8) (Tibshirani, 2011). Subsequently, we performed feature selection using the SVM-RFE algorithm in conjunction with 10-fold cross-validation, implemented via the “e1071” (version 1.7.16) and “caret” (version 7.0.1) R packages (Huang et al., 2014). Thereafter, the Random Forest (RF) algorithm was conducted with the “randomForest” (version 4.7.1.2) R package (Blanchet et al., 2020). To build the predictive model, parameters were optimized through 10-fold cross-validation. Then we assessed model performance based on the ROC curve and confusion matrix. Furthermore, gene importance was evaluated, and the top 10 highest-ranked genes were selected for subsequent analysis. Venn diagram analysis was employed to identify the overlapping genes derived from each algorithm in the PD and PTB datasets. These cross-talk genes were subsequently designated as candidate biomarkers for the two diseases.

### 2.6 Expression analysis and ROC curve analysis of candidate biomarkers

Using the “ggplot2” package, we examined the expression levels of the candidate biomarkers across control and disease groups (p < 0.05 for significance). With the “pROC” package (version 1.18.5), we carried out ROC curve analysis to elucidate the sensitivity and specificity of hub genes in the diagnosis of PD and PTB (Fayyad-Kazan et al., 2013; Hu et al., 2022), where an AUC value exceeding 0.5 was deemed indicative of diagnostic efficacy (Obuchowski and Bullen, 2018).

### 2.7 Functional enrichment analysis of common DEGs

Functional enrichment analysis was performed with the “clusterProfiler” R package (version 4.14.4), utilizing the Gene Ontology (GO) annotation system which encompasses three domains: cellular component (CC), biological process (BP), and molecular function (MF). We also performed Kyoto Encyclopedia of Genes and Genomes (KEGG) pathway analysis to complement our functional characterization by identifying significantly enriched biological pathways. Ultimately, we selected the top 10 most enriched terms using a p-value threshold of < 0.05 and generated a plot via the “ggplot2” package.

### 2.8 Immune cell infiltration analysis

To characterize immune cell infiltration in PD and PTB training datasets, we employed the single-sample gene set enrichment analysis (ssGSEA) utilizing the “GSVA” package (version 2.0.7) in R. The relative infiltration levels of various immune cells across samples were visualized using a heatmap. Immune cells with significant differences (P < 0.05) were further selected and presented in box plots to compare their infiltration levels between disease and control groups. To evaluate potential association, we assessed the relationship between immune cell infiltration and core gene expression using Spearman’s rank correlation and plotted the results in a heatmap.

### 2.9 Construction of PPI network

To construct a PPI network, the common DEGs were submitted to the STRING database (http://www.string-db.org/), a publicly accessible resource designed to predict both direct and indirect functional interaction between proteins based on integrated evidence and correlation metrics. We established a minimum interaction score threshold of 0.400 to construct the PPI network. Then we visualized the resulting interaction network using Cytoscape software (version 3.10.3). The “Stress” parameter was selected as it excels at quantifying inter-subnetwork bridging, which aligns with our goal of identifying cross-pathway hub genes, a choice supported by relevant network biology studies (Chin et al., 2014). Biologically, higher Stress values indicate a gene’s critical role in maintaining network connectivity, a key feature of disease-related hub genes (He and Zhang, 2006). Given that the candidate genes had been pre-screened through WGCNA and machine learning, only the “Stress” centrality parameter was computed, ranked, and visualized using the cytoHubba plugin in Cytoscape to evaluate their connectivity and confirm their key bridging roles within the PPI network.

### 2.10 Experimental validation

#### 2.10.1 Construction of the PD model

C57BL/6 wild-type mice were procured from the Experimental Animal Center with approval from the Animal Care and Use Committee of Anhui Medical University. The animals were maintained in an environment free of specific pathogens, with conditions maintained at a constant temperature of 24 °C ± 0.5 °C, 40%–70% relative humidity, and a 12:12-hour light/dark cycle. To achieve anesthesia, mice received an intraperitoneal administration of sodium pentobarbital (40 mg/kg). To establish an experimental PD model, the bilateral maxillary second molars of mice in the experimental group were ligated with 5–0 silk sutures for a duration of 4 weeks. Mice without ligature placement were designated as the control group. Subsequently, we harvested maxillary bone tissue samples from the mice to examine the expression levels of the core genes that had been identified as potential biomarkers.

#### 2.10.2 Western blot analysis

Total protein was first extracted from maxillary bone and heart tissues by lysing them in RIPA buffer containing protease and 1% phosphatase inhibitors. Subsequently, we measured the protein concentration using a commercial BCA kit (P0012, Beyotime Biotechnology). Protein separation was conducted using sodium dodecyl sulfate‐polyacrylamide gel electrophoresis, followed by transfer to PVDF membranes (Millipore). Following blocking with a primary antibody-specific solution, the membranes were probed with the specified primary antibodies: anti-β-actin (66009-1-Ig, Proteintech), anti-BIN2 (14245-1-AP, Proteintech), and anti-CD53 (85838-3-RR, Proteintech). Following visualization with the Western Bright ECL HRP substrate Kit, the relative expression levels of the target proteins were calibrated against β-actin.

#### 2.10.3 Real-time polymerase chain reaction (RT-PCR)

Total RNA was isolated from snap-frozen maxillary bone and heart tissues. The samples were first mechanically homogenized into a fine powder under liquid nitrogen and then processed using TRIzol Reagent (Invitrogen, USA). To assess gene expression, the mRNA expression levels of the target genes were quantified by quantitative PCR (SYBR Premix Ex Taq II), and the relative expression levels were analyzed by the 2^−ΔΔCT^ method with normalization to GAPDH. The following primer sequences were utilized in this study:

*Cd53*:Forward:5'-TCCAGACACAACTGCAGTGTTG-3’; Reverse:5'-GGAGTGAAACCACGATTTTGCC-3’.
*Bin2*:Forward:5'-CATTGTGGGGAACAATGACC-3’; Reverse:5'-GTAGTCTACCAGTTTCCGGC-3’.
*Gapdh*:Forward:5'-ATGGGTGTGAACCACGAGA-3’; Reverse:5'-CAGGGATGATGTTCTGGGCA.

#### 2.10.4 Statistical analysis

We performed the bioinformatics statistical analysis with R (v4.4.1) and carried out statistical testing using GraphPad Prism software. For comparisons between two groups, a two-tailed unpaired Student’s t-test was applied. For comparisons involving three or more groups, one-way ANOVA was utilized. All data are expressed as the mean ± standard deviation (SD). P < 0.05 was considered statistically significant.

## 3 Results

### 3.1 Data integration followed by batch effect correction

**FIGURE 2:**
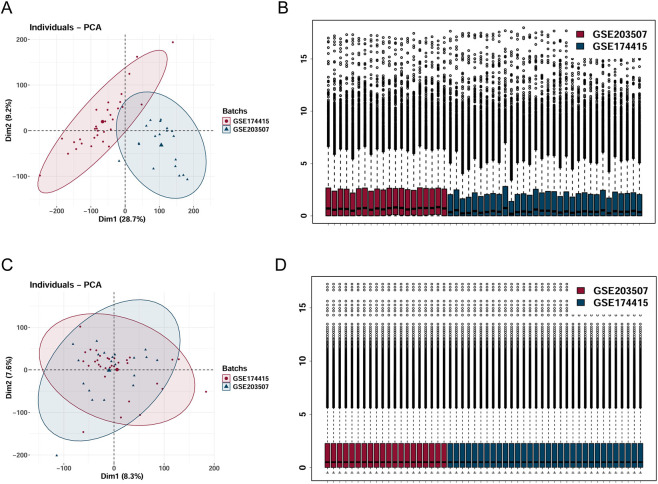
Merge the two datasets GSE174415 and GSE203507, and eliminate the batch effect. **(A,C)** PCA plots generated from the merged datasets, both before and after batch effect removal. **(B,D)** Boxplots generated from the merged datasets, both before and after batch effect removal.

We extracted and integrated placental tissue samples from GSE203507 and GSE174415. Following batch effect correction, a combined PTB dataset was obtained. As demonstrated in Figures 2A,B, the two PTB datasets displayed substantial disparities (GSE203507 and GSE174415). Figures 2C,D illustrated that the batch effects within the merged dataset were markedly mitigated. Through PCA and boxplot visualization, it was evident that the inter-dataset variations were significantly diminished.

### 3.2 Construction of the WGCNA network

**FIGURE 3:**
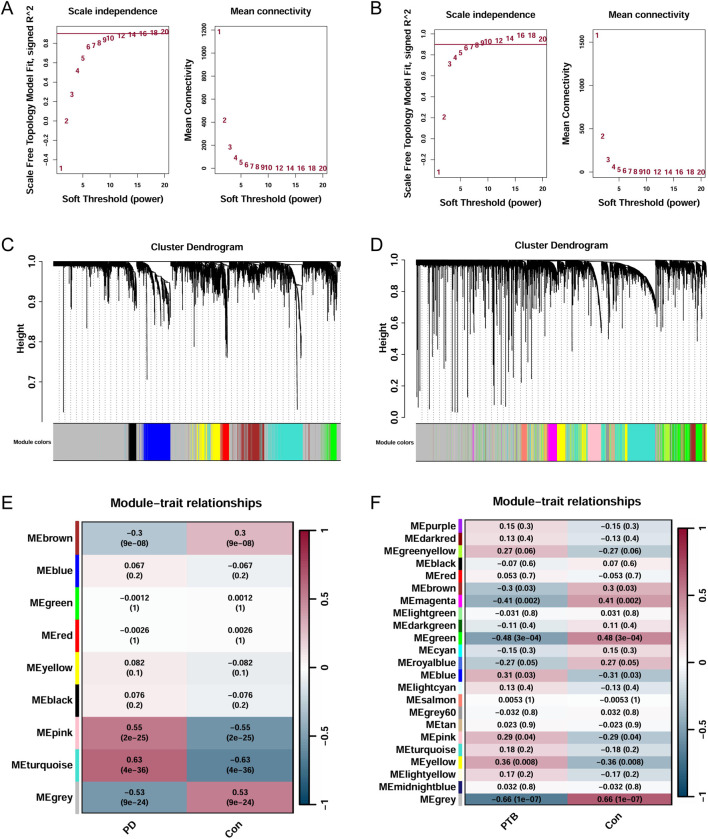
Weighted gene co-expression network analysis (WGCNA) in PD and PTB. **(A,B)** The selection of soft threshold in PD and PTB datasets. **(C,D)** Clustering dendrogram of co-expressed genes in PD and PTB datasets, with different modules in distinct colors. **(E,F)** Heatmap of the adjacency matrix among distinct modules in PD and PTB datasets.

WGCNA was used to identify the most significantly positively and negatively correlated modules in the PD and the combined PTB datasets. Soft thresholds of β = 16 and β = 8 were selected for the PD and the combined PTB datasets, respectively, to obtain a scale-free network topology (Figures 3A,B). We identified 9 co-expression modules in the PD dataset. The strongest positive correlation with PD was found in the turquoise module (r = 0.63, p = 4e-36), which contained 603 genes. In contrast, the brown module, with 342 genes, displayed the strongest negative correlation (r = −0.3, p = 9e-08) (Figures 3C,E). In the PTB analysis, 23 modules were detected. The yellow module, comprising 544 genes, displayed the highest positive correlation coefficient (r = 0.36, p = 0.008). The green module was identified as the most negatively correlated (r = −0.48, p = 3e-04) and included 451 genes (Figures 3D,F). Correlations between gene significance and module membership in the respective modules were visualized by scatter plots: the turquoise and brown modules for PD, and the yellow and green modules for PTB (Supplementary Figures S1A–D). Finally, a total of 44 common genes were identified from the module genes screened in both diseases by Venn diagram analysis.

### 3.3 Identification of DEGs

**FIGURE 4:**
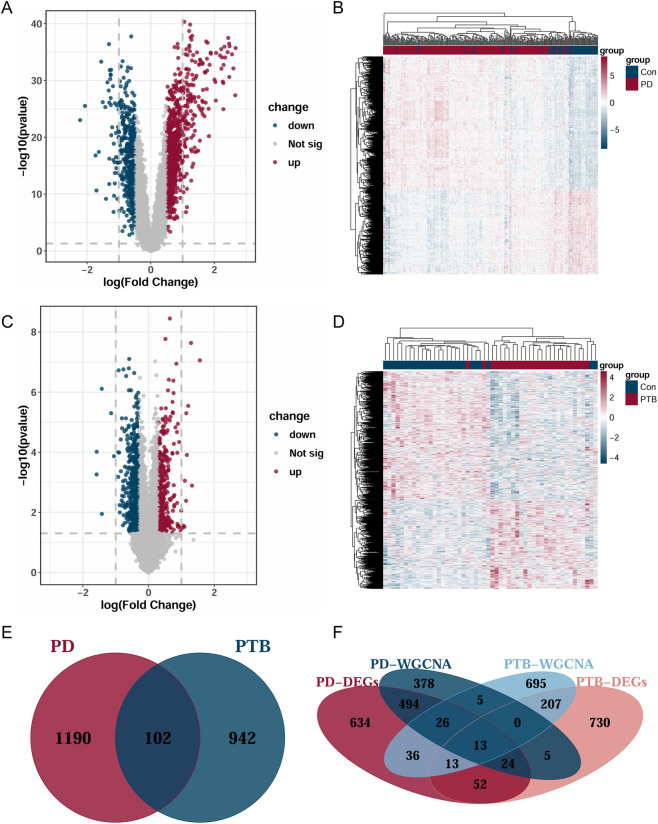
Differential expression analysis of PD and PTB. **(A,C)** Volcan plots presenting the DEGs in PD and PTB datasets. **(B,D)** The heatmap presenting the DEGs in PD and PTB datasets. **(E)** Venn diagrams presenting the number of common DEGs in PD and PTB datasets. **(F)** Venn diagrams presenting the number of shared genes in PD and PTB datasets.

We performed differential gene expression analysis on PD and combined PTB datasets. The PD analysis revealed 499 downregulated and 793 upregulated genes, while the PTB analysis identified 619 downregulated and 425 upregulated genes. To visualize these findings, the expression patterns of the DEGs were plotted on heatmaps for each dataset (Figures 4B,D). Additionally, we employed volcano plots to illustrate the statistical distribution of the DEGs in PD (Figure 4A) and PTB (Figure 4C). We obtained 102 common DEGs between PD and PTB using Venn diagrams (Figure 4E). Further analysis revealed a set of 13 shared genes between the WGCNA common genes and the common DEGs (Figure 4F).

### 3.4 Machine learning for identification candidate genes

**FIGURE 5:**
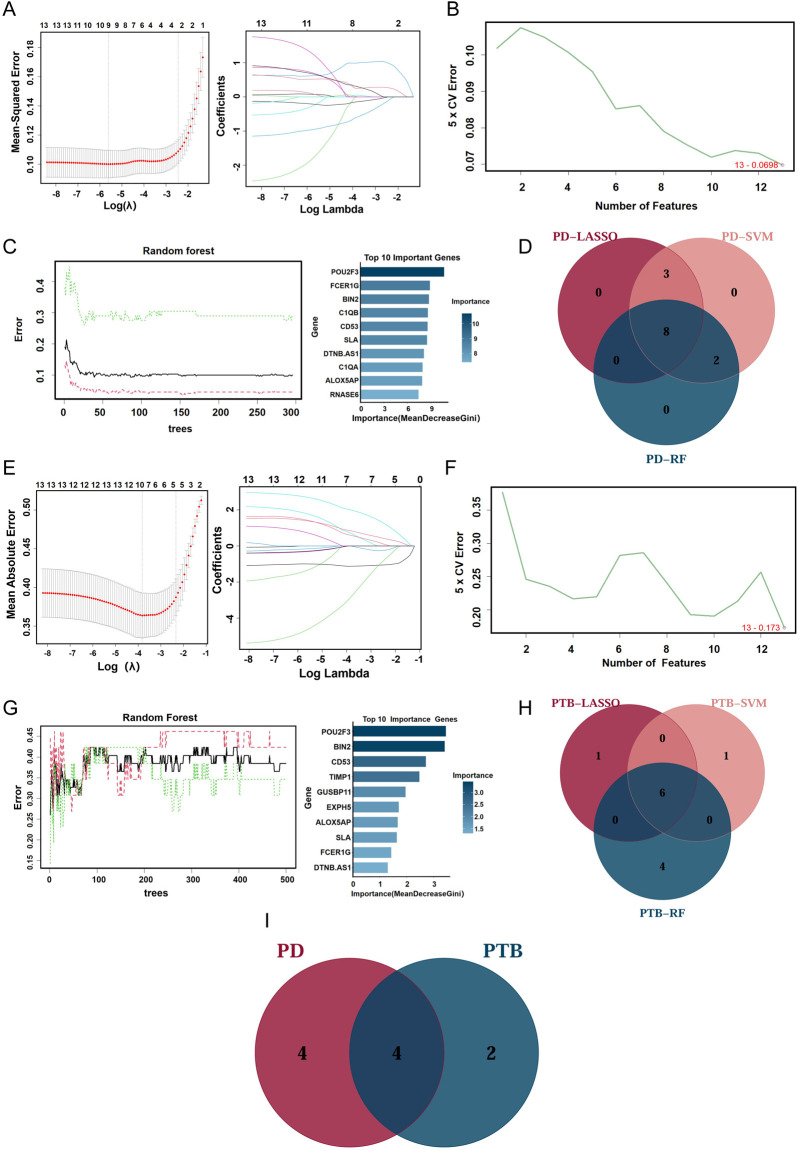
Three machine learning methods were utilized to identify cross-talk genes. **(A,E)** Biomarker screening based on the Lasso regression algorithm in PD and PTB datasets. The curve’s lowest point corresponded to the optimal gene count. Each curve in LASSO coefficient diagram represented one specific gene. **(B,F)** Biomarker screening based on the SVM-RFE algorithm in PD and PTB datasets. **(C,G)** The correlation between the number of trees and the error rates was presented in random forest. And the top 10 genes ranked by the importance scores from the random forest algorithm were selected. **(D,H)** Venn diagrams presenting the number of common genes obtained by three machine learning algorithms in PD and PTB datasets. **(I)** Venn diagrams presenting the number of candidate biomarkers in PD and PTB datasets.

For the identification of potential candidate biomarkers from the 13 shared genes, three machine learning algorithms were utilized. In the PD dataset, LASSO regression selected 11 genes (Figure 5A), while 13 genes achieving the lowest root mean square error (RMSE) were identified through the SVM-RFE algorithm (Figure 5B). The RF classifier listed the top 10 most important genes (Figure 5C). Eight overlapping genes were consistently identified in the PD group via all three methods (Figure 5D). Similarly, for the combined PTB dataset, LASSO regression selected 7 genes, SVM-RFE identified 13 genes, and the RF classifier highlighted 10 genes based on importance scores (Figures 5E–G). Subsequently, 6 overlapping genes were identified from the PTB group (Figure 5H). Ultimately, through Venn diagram analysis, four genes—FCER1G, CD53, POU2F3 and BIN2—were confirmed as candidate biomarkers (Figure 5I).

### 3.5 Expression analysis and ROC curve analysis of candidate biomarkers

**FIGURE 6:**
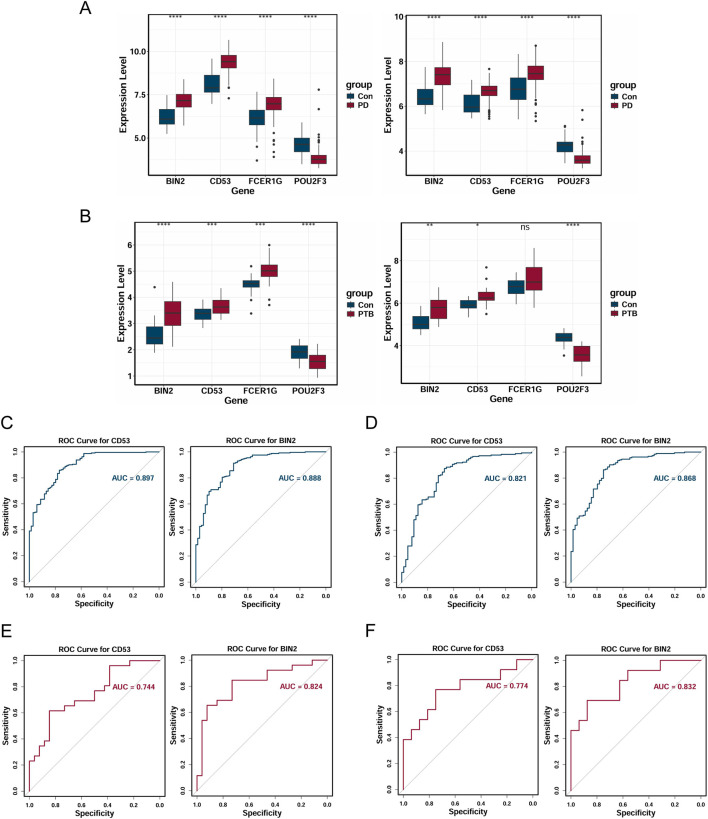
Validation of the expression level and diagnostic efficacy. **(A,B)** The expression level of cross-talk genes in PD training dataset and validation dataset **(A)**, in PTB training dataset and validation dataset **(B)**. **(C–F)** The ROC curves of candidate biomarkers in PD training dataset **(C)**, in PD validation dataset **(D)**, in PTB training dataset **(E)**, in PTB validation dataset **(F)**. (*P < 0.05; **P < 0.01; ***P < 0.001; ****P < 0.0001).

To evaluate the potential of FCER1G, CD53, POU2F3, and BIN2 as diagnostic biomarkers for PD and PTB, and to assess their diagnostic accuracy, we additionally incorporated two external validation sets from independent cohorts: the GSE10334 dataset for PD and a merged dataset (GSE18809, GSE73685, and GSE120480) of placental samples for PTB. We then assessed and visualized the expression level of these candidate biomarkers in both the training and validation cohorts using box plots. Figures 6A,B demonstrated markedly elevated expression of CD53 and BIN2 in the disease groups compared to the controls. To further analyze diagnostic efficacy, ROC curves for CD53 and BIN2 were plotted. The AUCs in the PD training dataset were 0.897 for CD53 and 0.888 for BIN2 (Figure 6C). Within the PD validation dataset, AUC values reached 0.821 and 0.868 for CD53 and BIN2, respectively (Figure 6D). For the PTB training dataset, the AUCs reached 0.744 (CD53) and 0.824 (BIN2), while in the PTB validation dataset, values were recorded as 0.774 and 0.832, respectively (Figures 6E,F). In summary, these results indicated that CD53 and BIN2 represented promising diagnostic biomarkers for both PD and PTB.

### 3.6 Functional enrichment analysis of common DEGs

**FIGURE 7:**
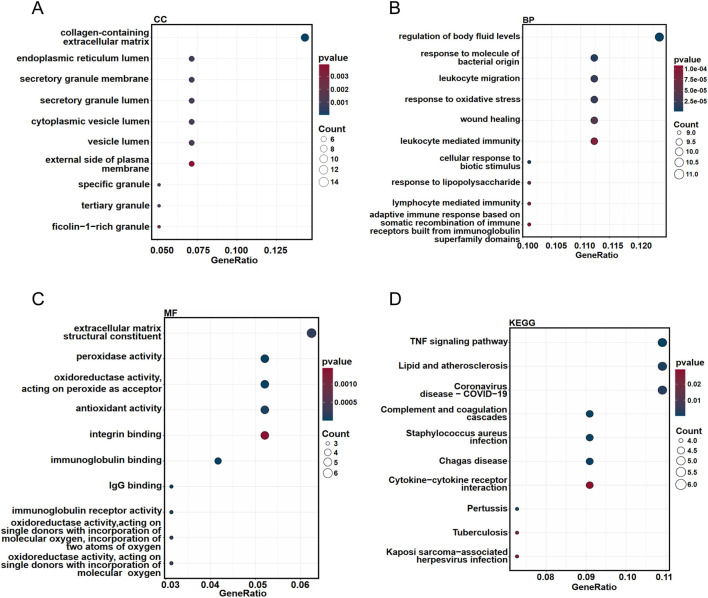
Functional enrichment analysis of common DEGs in PD and PTB. **(A–C)** GO categories of CC, BP, and MF. The top 10 of each were displayed. **(D)** KEGG enrichment analysis of common DEGs. Top 10 terms were displayed.

Functional enrichment analyses, including GO and KEGG pathway assessments, were performed to elucidate the biological roles of the common DEGs associated with both PD and PTB. CC analysis revealed significant associations with terms such as specific granule, collagen-containing extracellular matrix, and secretory granule membrane, among others (Figure 7A). Within BP, the predominant terms included response to molecule of bacterial origin, leukocyte migration and regulation of body fluid levels, etc (Figure 7B). For MF, key enrichments were identified in extracellular matrix structural constituent, peroxidase activity, and immunoglobulin binding, among other functions (Figure 7C). KEGG pathway analysis further showed notable enrichment in complement and coagulation cascades, lipid and atherosclerosis, and the TNF signaling pathway, along with other pathways (Figure 7D). Overall, these functional analyses showed that shared mechanism between PD and PTB may prominently involve inflammatory and immune responses.

### 3.7 Immune cell infiltration analysis

**FIGURE 8:**
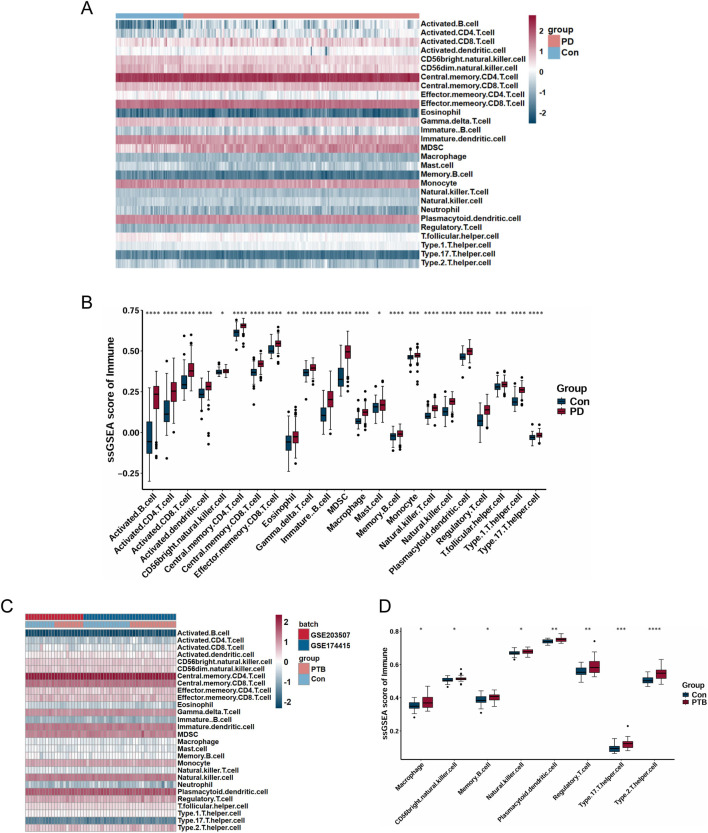
Analysis of immune cell infiltration. **(A,C)** Heatmap of immune infiltration analysis in PD and PTB datasets. **(B,D)** Boxplots showing the expression of each immune cell between disease and control. (*P < 0.05; **P < 0.01; ***P < 0.001; ****P < 0.0001).

Given that both PD and PTB share immune dysregulation as core pathological features linked to their pathogenesis (Hajishengallis, 2015; Bhati et al., 2023), and considering the significant enrichment of common DEGs in inflammatory and immune-related pathways, we further employed ssGSEA to characterize immune cell infiltration patterns and elucidated the immune regulation modes in PD and PTB training datasets. The diverse distribution of immune cells in each sample is illustrated in Figures 8A,C. Relative to controls, PD samples showed a significant increase in multiple cell types, including activated CD8 T cells, activated dendritic cells, central memory CD4 T cells, activated CD4 T cells, eosinophils, activated B cells, mast cells, macrophages, CD56bright natural killer cells, memory B cells, monocytes, central memory CD8 T cells, effector memory CD8 T cells, immature B cells, gamma delta T cells, natural killer T cells, regulatory T cells, plasmacytoid dendritic cells, T follicular helper cells, natural killer cells, MDSCs, type 1 T helper cells, and type 17 T helper cells (Figure 8B). Similarly, PTB tissues exhibited higher levels of memory B cells, regulatory T cells, CD56 bright natural killer cells, type 17 T helper cells, plasmacytoid dendritic cells, macrophages, natural killer cells, and type 2 T helper cells (Figure 8D). Thus, comparative analysis revealed a consistent upregulation across both diseases in the following immune subsets: natural killer cells, CD56bright natural killer cells, macrophages, memory B cells, plasmacytoid dendritic cells, regulatory T cells, and type 17 T helper cells.

Furthermore, correlation analyses illustrated via heatmap demonstrated significant correlation between various immune cells and the two core genes (Supplementary Figures S2A,B). Specifically, regulatory T cells and macrophages exhibited positive correlations with both CD53 and BIN2 in PD and PTB samples.

### 3.8 Construction of PPI network

**FIGURE 9:**
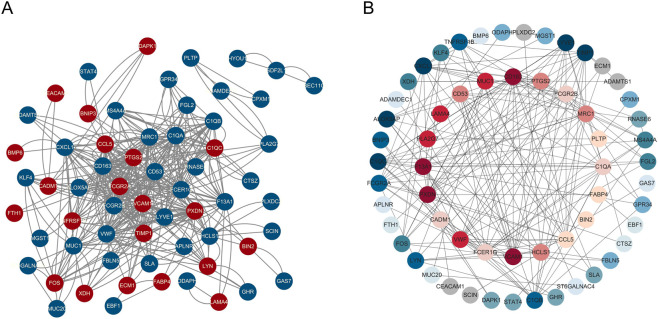
Construction of the PPI network and identification of hub genes. **(A)** PPI network associated with the common DEGs acting as nodes in a PPI network. **(B)** The common DEGs acting as nodes in a PPI network ranked by Stress. The top 20 genes were shown in the center of the network, with darker colors indicating higher rankings.

To further explore the functional interactions, we established a PPI network utilizing the STRING database with 102 common DEGs related to both PD and PTB. Following the removal of disconnected proteins, the final network contained 99 nodes and 202 edges, with nodes corresponding to proteins encoded by the DEGs and edges representing functional or physical interactions between these proteins (Figure 9A). We further analyzed the network utilizing Cytoscape software and various algorithms available in the “cytohubba” plugin. This included computation of Stress centrality values and gene ranking. The top twenty genes were positioned in the inner circle, while the remaining genes were distributed in the outer circle, with node color intensity gradually decreasing corresponding to their descending rank (Figure 9B). Final analysis identified CD53 and BIN2 as ranking 10th and 17th respectively in Stress centrality, indicating their high connectivity within the PPI network.

### 3.9 Experimental validation

**FIGURE 10:**
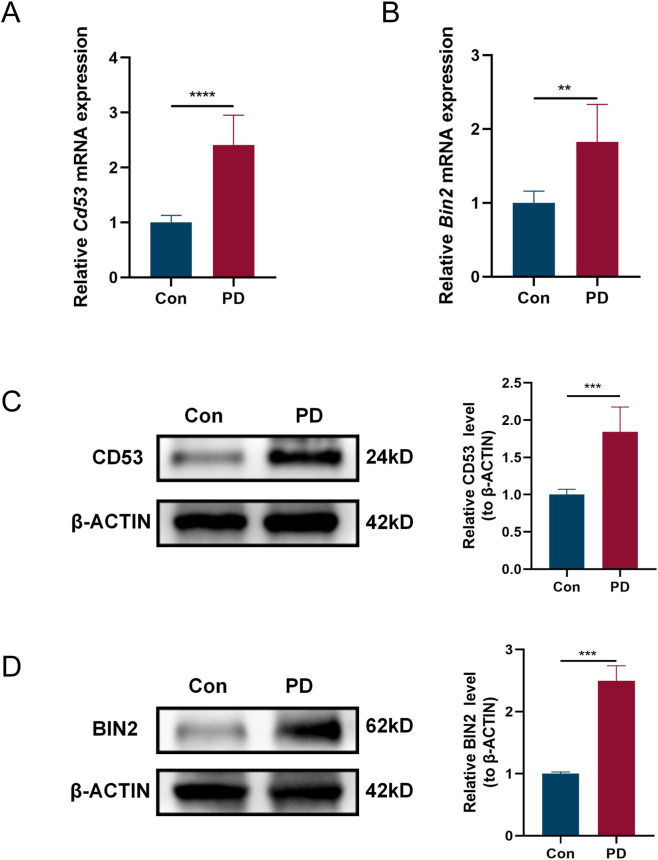
Validation of hub genes. **(A,B)** Relative mRNA expression levels of *Cd53* **(A)** and *Bin2* **(B)** in maxillary bone tissues of PD and control mice (n = 6 per group). **(C,D)** Relative protein expression levels of CD53 **(C)** and BIN2 **(D)** in PD and control mice (n = 3 per group). (**P < 0.01, ***P < 0.001, ****P < 0.0001).

To this end, we employed a ligature-induced PD model to further validate the expression pattern of the core genes. A concerted upregulation of CD53 and BIN2 was observed in the maxillary bone of PD group relative to control, as demonstrated by RT-qPCR (Figures 10A,B) and Western blotting (Figures 10C,D).

## 4 Discussion

While epidemiological association between PD and PTB is thoroughly confirmed, the common molecular mechanism mediating their co-occurrence remains incompletely understood. Although several hypotheses regarding shared pathways have been proposed, these mechanisms have yet to be fully validated. Bioinformatics serves as a powerful tool for elucidating the pathophysiology of diseases through genetic-level analysis. An integrated approach combining bioinformatics and machine learning was employed in this study, providing new insights into the pathogenesis of PD and PTB.

Utilizing integrated analysis of WGCNA analysis, differentially expressed genes, and three machine learning algorithms (LASSO, SVM-RFE, and RF), we ultimately identified two promising diagnostic biomarkers (CD53 and BIN2) between PD and PTB. A marked increase in the expression of both hub genes was detected in patient cohorts compared to healthy controls within both the training and validation sets. Furthermore, ROC curve analysis indicated their capacity to act as diagnostic biomarkers for PD and PTB, suggesting a vital function in the pathogenesis and effective diagnosis of both diseases.

CD53 (also known as OX44 or TSPAN25), a member of the tetraspanin superfamily, is predominantly expressed in various immune cells, such as B cells, T cells, myeloid cells, and others (de Winde et al., 2015; Wright et al., 1993). Previous studies have suggested its potential involvement in regulating the activation, co-stimulation, proliferation, and other functions of B cells and T cells through modulating immune cell adhesion, migration and intracellular signal transduction (Carmo and Wright, 1995; Demaria et al., 2020; Dunlock, 2020; Dunlock et al., 2022; Rasmussen et al., 1994; Rocha-Perugini et al., 2014; Tarrant et al., 2002). Studies have shown that CD53 is linked with local inflammation of periodontal tissue (Yin et al., 2024), which indicates a potential connection between CD53 and PD. Moreover, emerging evidence suggests an association between CD4^+^ T cells and PTB. Experimental studies have identified CD4^+^ T cells in fetal cord blood that are responsive to maternal alloantigens. These cells can induce myometrial contractility, revealing a novel mechanism underlying spontaneous PTB (Gomez-Lopez et al., 2022). Thus, it is hypothesized that CD53 may contribute to PTB via CD4^+^ T cells. Collectively, CD53 may be involved in the shared pathogenesis of PD and PTB through immunomodulatory mechanisms.

Bridging integrator 2 (BIN2) is highly expressed in leukocytes and correlated with key processes of leukocyte such as adhesion, migration, antigen uptake, and phagocytosis (Sánchez-Barrena et al., 2012). A previous study has documented the downregulation of BIN2 in neutrophils from patients with PD and PE. This suggests that it potentially plays a role in the shared pathophysiological mechanisms of the two diseases through immune dysregulation (Ruan et al., 2025). Placental dysfunction, a hallmark of early-onset PE, is a major contributing factor for PTB. So, it is pointed out that PE may induce PTB in pregnant women (Mukherjee et al., 2021; Weit et al., 2020). Huang et al. have identified BIN2 as a hub gene in fetal growth restriction, which is a condition associated with PTB (Garite et al., 2004; Huang et al., 2025). Therefore, BIN2 might serve as a key crosstalk gene between PD and PTB.

Due to the technical challenges in establishing a reliable PTB model, current research on the link between PD and PTB remains largely epidemiological without experimental validation. Therefore, we only employed a ligature-induced PD model to validate the expression of CD53 and BIN2. The results demonstrated that both core genes were consistently upregulated in the maxillary bone of PD mice. This key finding provides experimental support for the existing bioinformatics research connecting the two conditions.

GO and KEGG analyses showed that the common DEGs in PD and PTB were substantially enriched in terms mainly related to immune responses, including leukocyte migration, response to molecule of bacterial origin, specific granule, immunoglobulin binding, the complement and coagulation cascades, and the TNF signaling pathway. Periodontopathic bacteria release chemicals that stimulate both the innate and adaptive immune systems, which subsequently causes a sustained inflammatory response. This process results in the overexpression of proinflammatory cytokines and chemokines and the destruction of periodontal tissue (Ramadan et al., 2020; Silva et al., 2015). Certain inflammatory factors can further enter the bloodstream and contribute to systemic inflammation (Zhang and Lin, 2020), which could significantly increase the risk of PTB. Ebersole et al. utilized ligature-induced PD models in non-human primates to study its impact on pregnancy outcomes. The findings demonstrated that PD may elevate the risk of APOs, potentially through the mediation of systemic inflammation resulting from the release of pro-inflammatory mediators (Ebersole et al., 2014). Immunosuppression during pregnancy may reduce the host’s capacity to clear periodontal pathogenic bacteria by altering level of inflammatory mediators. This process can increase the susceptibility of pregnant women to PD. Elevated level of the bleeding index (BI) and 8-hydroxy-2′-deoxyguanosine (8-OHdG) which are inflammation-related indicators associated with PTB, may also indirectly suggest the presence of PD (Ye et al., 2021). In summary, studies indicate that the regulation of immune responses may represent a potential link connecting PD and PTB. The majority of experts believe that oral health issues during pregnancy not only affect the mother’s health but also impact the fetal oral development and the maturation of the immune system through various mechanisms (Zhang et al., 2025). Therefore, for women of reproductive age with PD, treatment of PD and control of associated inflammation during pre-pregnancy or pregnancy may reduce the risk of PTB.

Based on these findings, we additionally conducted an immune infiltration analysis to search for the immune cells that potentially contributed to the common pathogenesis of PD and PTB. Ultimately, we identified seven types of immune cells that were upregulated in both the PD and PTB disease groups: natural killer cells, CD56bright natural killer cells, macrophages, memory B cells, plasmacytoid dendritic cells, regulatory T cells, and type 17 T helper cells. Natural killer cells promote inflammation and enhance periodontal bone resorption by releasing pro-inflammatory cytokines including IFN-γ and by direct interactions with periodontal pathogens (Wilensky et al., 2015). CD56 bright natural killer cells are closely related to PD because they are a type of natural killer cell which are primarily characterized by their immunoregulatory function. Macrophages are found in large quantities in the gingival tissue and gingival crevicular fluid of PD patients (Kang et al., 2016). Furthermore, Cekici et al. demonstrated a direct relationship between the levels of macrophage infiltration and PD severity (Cekici et al., 2014). Memory B cells which are located in the connective tissue of healthy gingiva play a potential role in sustaining periodontal homeostasis. A higher count of memory B cells is a sign that PD is improving (Mahanonda et al., 2016). Studies indicated that plasmacytoid dendritic cells, capable of producing type I interferon, exhibit a complex causal relationship with PD (Ye et al., 2024). The frequency of regulatory T cells, which can suppress immune responses and protect alveolar bone, is reduced in PD, leading to their protective function compromised (Han et al., 2018). Type 17 T helper cells can promote bone resorption and inflammatory responses through the secretion of cytokines such as IL-17. Thus, they serve as key players in the osteoimmunology of PD (Lü and Wen, 2020). It has been found that a distinct subset of natural killer cells characterized by CD56bright expression exists in the uterus. These cells contribute to pregnancy establishment and placentation by regulating trophoblast invasion, spiral artery remodeling, and endometrial decidualization. Thus, it serves as critical immune players in the maintenance of early pregnancy (Kanter et al., 2021). Macrophages may participate in PTB pathogenesis though multiple mechanisms, including the increased infiltration, polarization towards the M1 pro-inflammatory subtype, and the release of matrix metalloproteinases which could degrade cervical collagen and disrupt tissue integrity (Yao et al., 2019). Minor modifications in memory B cells may be an epiphenomenon of chronic inflammation at the maternal-fetal interface, but not a core driver of PTB (Leng et al., 2019). Regulatory T cells surrport normal pregnancy progression through helping maintains immune tolerance at the maternal-fetal interface and suppressing excessive inflammatory responses. A reduction in their numbers or functional impairment can disrupt this immune equilibrium and then potentially result in PTB (Mureanu et al., 2024). Type 17 T helper cells may induce or promote PTB by disrupting the type 17 T helper cell/regulatory T cell ratio balance and exacerbating maternal inflammatory responses (Figueiredo and Schumacher, 2016). However, the role of plasmacytoid dendritic cells in PTB remains unclear. Taken together, targeting the regulation of abundance and function of specific immune cell populations, such as natural killer cells, CD56bright natural killer cells, macrophages, regulatory T cells, and type 17 T helper cells may lead to novel interventions that suppress the development of PD and PTB.

Nevertheless, there are also several limitations in this study. Firstly, the sample sizes of the datasets we used were limited, especially the PTB-related data. This is primarily due to the scarcity of datasets related to human samples in the GEO database. Having access to a larger sample size of PTB-related datasets would make our future findings more robust. Furthermore, the precise roles of CD53 and BIN2 require further validation through additional *in vivo* and *in vitro* functional experiments. In relevant animal models, phenotypic and functional assessments following targeted gene manipulation will help confirm their *in vivo* biological significance. Finally, the analysis in the current study lacked data from patients with comorbid PD and PTB. Future studies would benefit from including cohorts with concurrent diagnoses. Despite these limitations, the initial identification of these two potential biomarkers for PD and PTB may help raise new hypotheses and provide a foundation for further mechanistic research.

## 5 Conclusion

In this study, we integrated bioinformatics and machine learning approaches to identify CD53 and BIN2 as potential biomarkers for PD and PTB, and they may be involved in the crosstalk between the two diseases through immune pathways. Furthermore, expression analysis and ROC curve analysis confirmed the favorable diagnostic performance of both CD53 and BIN2. In conclusion, this study establishes a theoretical foundation for further exploring the common pathogenic mechanism of PD and PTB from multidimensional perspectives including genetics, and immune infiltration, and provides novel insights for the prevention and diagnosis of both diseases.

## Data Availability

The original contributions presented in the study are included in the article/Supplementary Material, further inquiries can be directed to the corresponding authors.
