# Supplementary material for: Integrated bioinformatics and machine learning to explore the common mechanisms and potential biomarkers between periodontitis and preterm birth
Source: Front Cell Dev Biol. 2026 Feb 20;14:1763374. doi: 10.3389/fcell.2026.1763374 (PMC12963270; doi:10.3389/fcell.2026.1763374)
Supplement: Supplementary file 1 [file DataSheet1.docx]

Supplementary Material

# Supplementary Figures


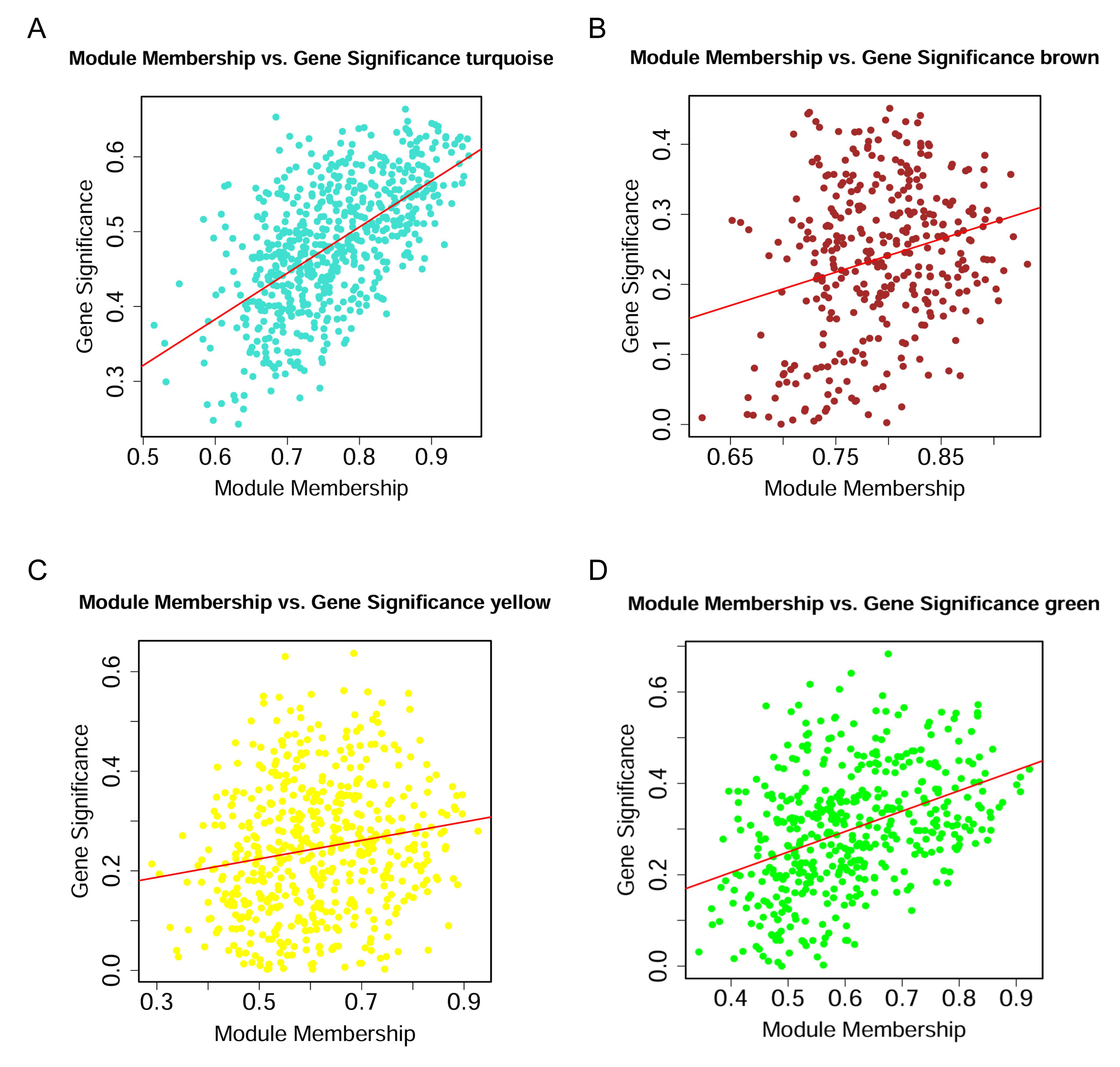


**Supplementary Figure 1.** The link between gene significance and module membership in the WGCNA network. (A) Scatter plot of gene significance against module membership for the turquoise module in the PD dataset (GSE16134). (B) Scatter plot of gene significance against module membership for the brown module in the PD dataset (GSE16134). (C) Scatter plot of gene significance against module membership for the yellow module in the PTB combined dataset. (D) Scatter plot of gene significance against module membership for the green module in the PTB combined dataset.


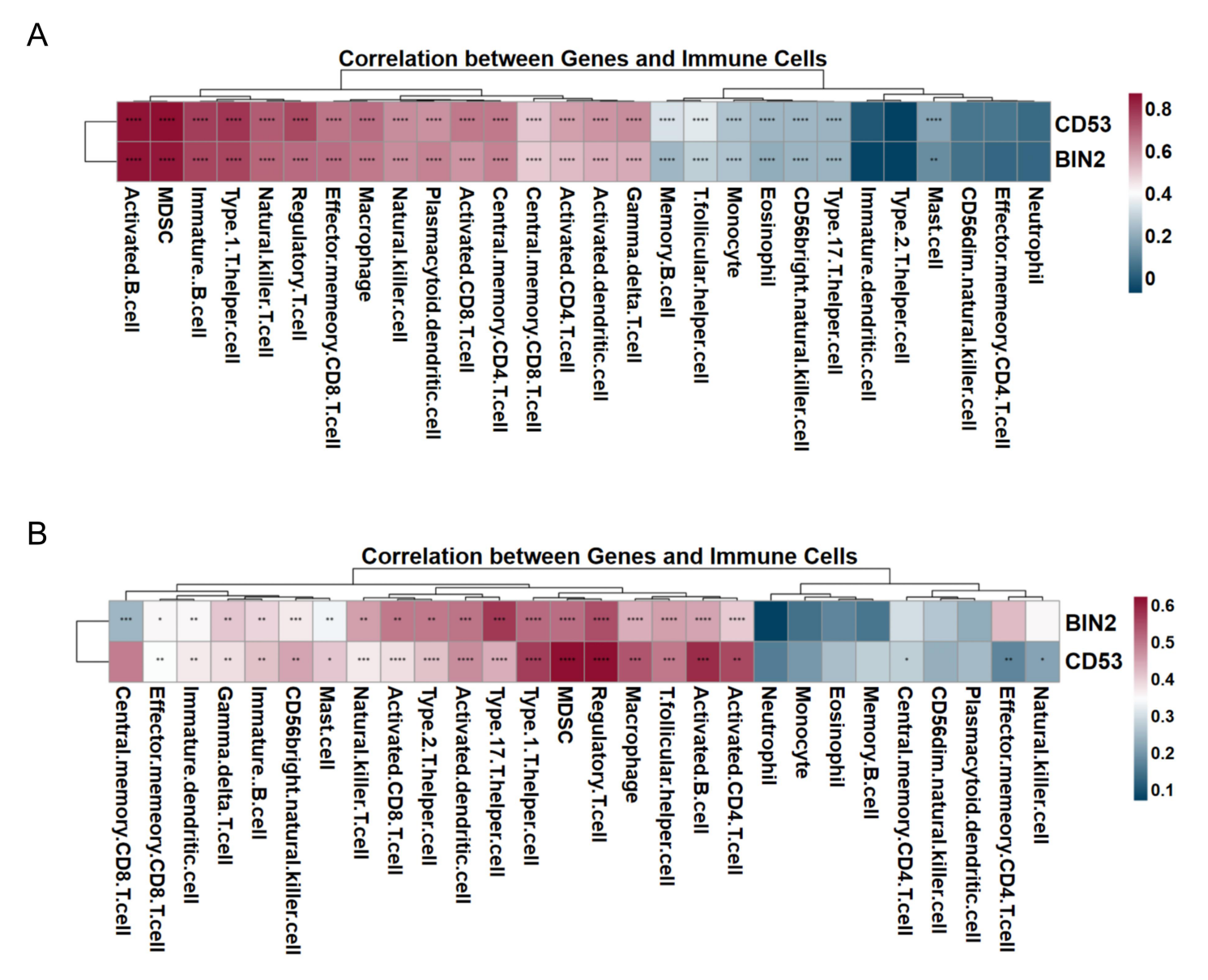


**Supplementary Figure 2.** Correlation analysis of potential biomarkers with immune cell infiltration. (A) Correlation between 2 potential biomarkers and immune cell infiltration in the PD dataset (GSE16134). (B) Correlation between 2 potential biomarkers and immune cell infiltration in the PTB combined dataset. (**P*<0.05; ***P*<0.01; ****P*<0.001; *****P*<0.0001)
